# Evaluation of Safe Firearm Storage Messaging in a Sample of Firearm-Owning US Military Service Members

**DOI:** 10.1001/jamanetworkopen.2022.35984

**Published:** 2022-10-11

**Authors:** Michael D. Anestis, Craig J. Bryan, Daniel W. Capron, AnnaBelle O. Bryan

**Affiliations:** 1 New Jersey Gun Violence Research Center, Piscataway; 2 Rutgers, the State University of New Jersey, Piscataway; 3 The Ohio State University Wexner Medical Center, Columbus; 4 University of Southern Mississippi, Hattiesburg

## Abstract

**Importance:**

Nearly two-thirds of military suicides involve firearms, and safe firearm storage is rare.

**Objective:**

To examine whether US military service members endorse greater openness to safe firearm storage depending on the content of the visual message they are randomly assigned to view.

**Design, Setting, and Participants:**

This comparative effectiveness study used a 3 × 2 × 2 factorial design to randomize US military service members to view 1 of 12 visual messages on safe firearm storage. Willingness to use safe firearm storage practices was assessed immediately before and after exposure to the message. Participants were recruited using the KnowledgePanel Calibration approach. Inclusion criteria included current membership in the US military and current firearm ownership. The KnowledgePanel sample was fielded from December 3 to 27, 2021, with a 76% completion rate and 45 individuals determined to be qualified (28% qualification rate). The opt-in sample was fielded December 7, 2021, through January 4, 2022, with 699 individuals (3%) qualified and 674 included in the final data set.

**Exposures:**

Messages shared the same image and text on safe firearm storage but varied in messenger occupation (eg, primary care physician, security forces, or combat controller), the presence of text validating the perspective of firearm owners, and the presence of text validating the drive for home protection.

**Main Outcomes and Measures:**

Outcomes included changes in willingness to use 4 at-home (unloaded, separate from ammunition, in a locked location, and with a locking device) and 3 away-from-home (with family or friend, at a firearm retailer, or at a law enforcement agency) firearm storage practices. All analyses, including sample descriptives, are based on weighted data.

**Results:**

Of the 719 individuals in the data set, 367 (median [range] age, 33.64 [18-86] years; 80.4% male; 71.4% White) who endorsed not currently storing firearms using the methods assessed were included in analyses. In a multivariate analysis of variance, a significant interaction was found among time, messenger profession, gun-friendly text, and home protection text across all outcomes (Wilks’ λ *F* = 2.09; *P* = .01; _p_η^2^ = 0.040); however, in a post hoc repeated-measures analysis of variance, the interaction was statistically significant only for storing firearms away from home with a trusted family member or friend (*F* = 5.42; *P* = .005; _p_η^2^ = 0.030). The profession of the messenger was more consistently associated with shifts in willingness than was the message content, although this varied across storage options.

**Conclusions and Relevance:**

The findings of this comparative effectiveness study suggest that several combinations of messenger and content may be associated with willingness to endorse safe firearm storing practices, with particularly consistent positive findings for messages featuring security forces. The scalability and dosage potential of this intervention may render visual messaging valuable for promoting safe firearm storage at the population level.

## Key Points

**Question:**

Is the content of visual messaging associated with willingness to adopt safe firearm storage practices?

**Findings:**

In this comparative effectiveness study of 367 US military service members who did not currently endorse safe firearm storage practices, several combinations of message components were associated with significant positive changes in openness to specific firearm storage practices. Positive change was most consistently seen for within-home storage options (eg, locking devices) rather than outside-of-home storage options (eg, at a firearm retailer).

**Meaning:**

These findings suggest that safe firearm storage messaging is a scalable intervention that could promote life-saving behavior.

## Teaser

This comparative effectiveness study of US military service members assesses whether visual messages about safe firearm storage are associated with openness to safe firearm storage practices.

## Introduction

Of the 45 979 individuals who died by suicide in the US in 2020, 24 292 (52.8%) died from gunshot wounds.^1^ Among active-duty military service members, 318 of the 498 suicide deaths (63.9%) in 2019 resulted from firearms.^2^ Studies have repeatedly demonstrated that firearm access is associated with suicide death^3,4,5,6^ and that risk is further increased when firearms are stored unsafely.^7^

Despite this association between firearms and suicide deaths, use of safe firearm storage is limited. Within a nationally representative sample of firearm owners (n = 3949),^8^ 33.3% of veteran firearm owners stored at least 1 firearm both loaded and unlocked, with substantial variability in storage practices based on demographic characteristics. Among military service members in primary care (n = 1652),^9^ 86% of firearm owners with recent suicidal thoughts (vs 58% without) stored their firearms loaded and unlocked. This finding was replicated within the US National Guard, with those endorsing lifetime suicidal thoughts 2.62 times more likely to store firearms loaded and unlocked.^10^

The lack of safe firearm storage among firearm-owning active-duty military service members and veterans highlights a failure in messaging. Some evidence supports the use of lethal-means counseling to promote meaningful changes in firearm storage behavior among military service members^11^; however, scalability of this intervention is limited, thereby precluding population-wide shifts in storage. Broader public health messaging thus may be a pivotal suicide prevention tool^12^; however, limited data exist to guide development and implementation.

Emerging research on firearm storage messaging has emphasized the importance of validating the perspective of firearm owners, including a tendency to value safety and home protection.^13,14,15^ Furthermore, studies^16,17,18^ examining preferred messengers have shown that firearm owners tend to view law enforcement officers, military service members, and veterans as credible messengers on safe firearm storage. Experimental data examining these considerations are lacking, however.

We recruited a large, nationally representative sample of firearm-owning military service members and randomized them to view different visual messages on safe firearm storage for suicide prevention. Each message shared core components; the same image, name of the messenger, and text regarding the need for safe firearm storage. The messages varied, however, on 3 components: (1) the profession of the messenger (primary care physician, security forces, or combat controller), (2) the presence of text validating the perspective of firearm owners (gun friendly), and (3) the presence of text validating the drive for home protection. We examined to what extent these message elements were associated with immediate change in willingness to use multiple firearm storage practices. Given limited relevant data, we frame these results as an exploratory and preliminary guide for the development of safe firearm storage messaging.

## Methods

### Participants and Procedures

Data for this comparative effectiveness study were collected by Ipsos using the KnowledgePanel (KP) Calibration approach.^19^ The KP sample was weighted to represent the current military population, and the KP participants were then leveraged to calibrate the opt-in participants to mirror that representativeness. Opt-in panels include Ipsos proprietary panels plus those from partner organizations. Opt-in panels were router panels, wherein panel members are allocated to surveys the system identifies them as being eligible for rather than organizations sending out email invitations for specific surveys (eAppendix in the Supplement). Participants provided informed consent by checking a box on an online informed consent document before beginning the study. All data were deidentified. All procedures were approved by the Rutgers Institutional Review Board and the US Army Medical Research and Development Command, Office of Research Protections, Human Research Protection Office (HRPO). This study followed the International Society for Pharmacoeconomics and Outcomes Research (ISPOR) reporting guideline.

Inclusion criteria included current membership in the US military and current firearm ownership. The KP sample was fielded December 3 to 31, 2021, with a 76% completion rate and 45 individuals determined to be qualified (28% qualification rate). The opt-in sample was fielded by Ipsos and their partner organizations from December 7, 2021, through January 4, 2022, with 699 individuals (3%) meeting the inclusion criteria. Of the 699 qualified opt-in participants, 674 were included in the final data set, with 25 removed based on responses to a low base rate free response item, resulting in a final sample size of 719. Demographic characteristics, including race and ethnicity, were self-reported in the survey by participants. Race and ethnicity data are collected routinely in KP profiles and were analyzed in this study to assess whether and how various aspects of identity may be associated with differing responses to various messages on safe firearm storage. All analyses, including sample descriptives, are based on weighted data. Only participants who did not endorse already storing their firearms safely (n = 367) were included in the analyses.

Weighting, including calibration, was performed for those who completed screening (eg, US service members regardless of firearm ownership status). Qualified cases then carried appropriate weights for this subpopulation. Because of the small base size, the calibration benchmarks were derived from KP profile data, not the KP participants in this specific study.

### Willingness to Use Firearm Storage Methods

Our outcome variables included participants’ willingness to use 7 different firearm storage practices. The options included 4 at-home storage approaches (unloaded, separate from ammunition, in a locked location, and with a locking device) and 3 away-from-home storage approaches (with a family member or friend, with a firearm retailer, or with a law enforcement agency). Participants were presented with a matrix of storage options and asked, “How willing—if at all—would you be to use the following firearm storage procedures to prevent your own suicide?” Responses were scored from 1 (“not at all open”) to 5 (“extremely open”). Participants who did not respond to storage items or who scored 6 (“I already store all of my firearms in this method”) were excluded from analyses. These variables were assessed immediately before (time 1) and immediately after (time 2) exposure to the stimulus.

### Visual Messages

Using a 3 × 2 × 2 factorial design, participants were randomized to view 1 of 12 visual messages (eFigures 1-12 in the Supplement) developed in consultation with the Defense Suicide Prevention Office. All 12 messages used the same image of a military service member in an armory, with the same name (Brian Robinson) assigned to the individual in the image. All images also included the same 2 base texts. The first base text said, “Suicide has been the number one cause of death for service members over the past three years and 60% of those deaths were by personal firearms.” The second base text said, “Simple steps can save lives. To prevent suicide, store your firearms unloaded, separate from ammunition, in a locked location, and store them away from home during times of stress.” The messages varied with respect to the occupation of the messenger (primary care physician, security forces, or combat controller), the presence of text validating the perspective of firearm owners (“As a firearm owner, you understand the importance of safety as well as anyone.”), and the presence of text validating the drive for home protection (“83% of firearm deaths in the military are due to suicide. There are several methods for keeping your home safe, including alarm systems and dogs.”). Combat controllers are deployed first into combat and hostile environments to establish position and provide support for units that follow. Security forces are trained in both law enforcement and combat tactics and protect both domestic and overseas bases. Of note, security forces are more likely than others to carry firearms while on base. The shoulder of the service member’s uniform was digitally altered for images identifying the messenger as a primary care physician to ensure accuracy. Randomization was stratified based on sex (male vs female) and defensive firearm ownership (yes vs no; 61% endorsed defensive firearm ownership).

### Statistical Analysis 

We used a repeated-measures multivariate analyses of variance (MANOVAs), which allows for the simultaneous consideration of multiple correlated dependent variables, thereby identifying patterns across dependent variables and increasing statistical power while reducing the joint error rate. Post hoc repeated-measures analyses of variance (ANOVAs) were then used to probe significant interactions for each dependent variable. Messenger profession, gun-friendly text, and home protection text served as the factors, and each of the 7 firearm storage behaviors (time 1 and time 2) served as outcome variables. To account for the relatively large number of comparisons, we used a false discovery rate of 0.05.^20^ An a priori power analysis indicated that, to have 0.8 power with a 2-tailed α = .05, we would need a sample size of 112 for a large effect size, 270 for a medium effect size, and 1634 for a small effect size. A 2-sided *P* < .05 was considered to be statistically significant.

## Results

Of the 719 individuals in the data set, 367 (median [range] age, 33.64 [18-86] years; 80.4% male and 19.6% female; 8.0% Asian, 20.5% Black, 10.4% Native American or Alaska Native, 2.5% Pacific Islander, 70.8% White, 2.1% of other race; and 33.6% of Hispanic/Latin[x] ethnicity) who endorsed not currently storing firearms using the methods assessed were included in analyses. Sample characteristics by condition are given in Table 1. Mean scores on openness to firearm storage practices at time 1 and time 2 by condition are given in Table 2. Of the 28 analyses involving exposure to a stimulus including the primary care physician, 9 (32.1%) resulted in mean decreases in openness to a specific storage practice. Those 9 decreases accounted for 60.0% of all decreases across messengers, with decreases ranging from 0.9% to 11.3%. In contrast, the security forces and combat controller stimuli each resulted in 3 mean decreases in openness to using specific firearm storage practices, with decreases ranging from 1.6% to 6.4% for the combat controller and 0.6% to 1.9% for the security forces.

**Table 1.** Characteristics of the Study Participants by Condition^a^

Characteristic	Condition											
	PCP, non–gun friendly, and no home protection (n = 35)	PCP, gun friendly, and no home protection (n = 32)	PCP, non–gun friendly, and home protection (n = 30)	PCP, gun friendly, and home protection (n = 30)	Security forces, non–gun friendly, no home protection (n = 42)	Security forces, gun friendly, and no home protection (n = 41)	Security forces, non–gun friendly, and home protection (n = 41)	Security forces, gun friendly, and home protection (n = 24)	Combat controller, non–gun friendly, and no home protection (n = 45)	Combat controller, gun friendly, and no home protection (n = 33)	Combat controller, non–gun friendly, and home protection (n = 25)	Combat controller, gun friendly, and home protection (n = 31)
Age, mean (SD), y	31.57 (5.41)	31.18 (8.03)	36.59 (11.33)	32.43 (12.55)	37.39 (13.19)	31.15 (8.85)	32.77 (12.55)	38.12 (10.22)	34.51 (11.10)	43.31 (16.09)	34.05 (9.11)	30.22 (9.98)
Sex												
Male	88.6	78.1	83.3	80.0	76.2	73.2	75.6	87.5	88.9	75.8	72.0	71.0
Female	11.4	21.9	16.7	20.0	23.8	26.8	24.4	12.5	11.1	24.2	28.0	29.0
Race and ethnicity												
Asian	3.1	6.3	3.4	0.0	9.4	28.1	6.3	0.0	0.0	9.4	15.6	18.8
Black	6.0	16.7	2.4	9.5	13.1	11.9	26.8	25.0	15.9	6.1	19.2	9.7
Native American or Alaska Native	17.6	25.0	17.2	0.0	2.4	20.8	11.4	0.0	11.4	15.2	24.0	0.0
Pacific Islander	0.0	6.3	0.0	0.0	4.8	2.4	0.0	0.0	0.0	9.1	0.0	8.0
White	73.5	62.5	82.8	63.3	69.8	48.8	68.3	76.0	84.1	66.7	68.0	87.1
Other	0.0	0.0	6.7	10.0	2.3	4.9	0.0	0.0	0.0	0.0	0.0	0.0
Ethnicity												
Hispanic or Latin(x)	28.6	46.9	27.6	26.7	30.2	34.1	51.2	20.0	29.5	12.1	52.0	45.2
US military branch												
Army												
Active duty	25.7	51.6	48.3	30.0	42.9	19.0	48.8	64.0	59.1	15.2	52.0	38.7
Reserve	2.9	6.5	6.9	16.7	14.0	9.8	4.9	12.0	2.3	24.2	20.0	6.5
National Guard	14.7	3.2	23.3	22.6	16.3	0.0	22.0	24.0	27.3	12.1	16.0	19.4
Air Force												
Active duty	17.1	16.1	10.3	16.7	30.2	19.5	9.8	4.0	13.6	18.2	20.0	22.6
Reserve	14.3	3.1	6.9	6.7	14.3	0.0	2.4	4.0	4.5	0.0	11.5	3.2
National Guard	0.0	0.0	0.0	6.7	16.3	7.3	7.3	0.0	2.2	9.1	0.0	3.2
Navy												
Active duty	17.1	25.0	16.7	20.0	7.0	9.8	2.4	8.0	4.5	18.2	4.0	9.7
Reserve	2.9	0.0	3.4	0.0	0.0	2.4	0.0	8.0	4.4	0.0	16.0	6.5
USMC												
Active duty	5.9	0.0	6.9	13.3	16.7	23.8	9.8	0.0	6.7	9.1	4.0	6.5
Reserve	5.7	0.0	17.2	10.0	0.0	0.0	0.0	4.0	0.0	0.0	0.0	3.2
Coast Guard												
Active duty	0.0	0.0	6.7	0.0	0.0	2.4	0.0	0.0	2.2	0.0	0.0	3.2
Reserve	2.9	0.0	3.3	9.7	0.0	0.0	0.0	0.0	13.3	3.0	0.0	0.0
PHS	5.7	3.1	6.5	10.0	0.0	12.2	4.8	0.0	0.0	3.0	16.1	0.0

Abbreviations: PCP, primary care physician; PHS, Public Health Service; USMC, US Marine Corps.

^a^ Data are presented as weighted percentages unless otherwise indicated.

**Table 2.** Mean Scores on Openness to Specific Firearm Storage Practices by Condition at Time 1 (T1) and Time 2 (T2)^a^

Practice	Condition											
	PCP, non–gun friendly, and no home protection (n = 35)	PCP, gun friendly, and no home protection (n = 31)	PCP, non–gun friendly, and home protection (n = 29)	PCP, gun friendly, and home protection (n = 30)	Security forces, non–gun friendly, and no home protection (n = 42)	Security forces, gun friendly, and no home protection (n = 41)	Security forces, non–gun friendly, and home protection (n = 41)	Security forces, gun friendly, and home protection (n = 25)	Combat controller, non–gun friendly, and no home protection (n = 44)	Combat controller, gun friendly, and no home protection (n = 33)	Combat controllers, non–gun friendly, and home protection (n = 25)	Combat controller, gun friendly, and home protection (n = 31)
Unloaded												
T1	3.63	3.00	3.27	3.08	3.28	2.73	3.12	2.82	2.88	3.41	3.53	3.31
T2	3.27	3.83	3.03	2.74	3.61	3.06	3.60	3.21	3.40	3.64	3.98	3.73
% Change	−9.9	+27.7	−7.3	−11.0	+10.1	+12.1	+15.4	+13.8	+18.1	+6.7	+12.7	+12.7
Separate ammunition												
T1	3.51	3.00	3.09	2.53	3.40	2.77	3.17	2.83	2.82	3.55	3.02	3.10
T2	3.47	4.09	2.74	2.67	3.38	2.88	3.41	3.03	3.54	3.57	3.38	3.80
% Change	−1.1	+26.3	−11.3	+5.5	−0.6	+4.0	+7.6	+7.1	+25.5	+0.6	+11.9	+25.8
Locked												
T1	2.91	3.02	3.31	2.45	3.19	2.68	3.31	3.36	2.77	3.70	2.96	2.96
T2	3.86	4.52	3.17	2.51	3.61	2.63	3.76	3.73	3.74	4.37	3.79	4.07
% Change	+32.6	+49.7	−4.2	+2.4	+13.2	−1.9	+13.6	+11.0	+35.0	+18.1	+28.0	+37.5
Locking device												
T1	3.07	3.32	3.34	2.63	2.79	2.53	3.64	3.33	2.62	4.00	2.98	3.44
T2	4.01	4.68	3.02	2.93	3.68	2.56	4.07	3.54	3.69	4.20	3.44	3.32
% Change	+30.6	+41.0	−9.6	+11.4	+31.9	+1.2	+11.8	+6.3	+40.8	+5.0	+15.4	−6.4
Family or friend												
T1	3.16	2.90	2.96	2.31	3.09	2.51	3.11	3.30	2.93	2.92	3.07	2.76
T2	3.13	3.83	2.97	2.88	3.29	3.00	3.64	3.80	3.75	2.85	3.02	3.36
% Change	−0.9	+32.1	+0.3	+24.7	+6.5	+19.5	+17.0	+15.2	+28.0	−2.4	−1.6	+21.7
Retailer												
T1	2.97	2.92	2.91	2.63	2.93	2.53	3.29	3.09	2.80	2.59	3.17	2.64
T2	3.02	3.49	2.51	2.86	3.03	2.78	3.69	3.31	3.60	3.44	3.29	3.06
% Change	+1.7	+19.5	−13.7	+8.7	+3.4	+9.9	+12.2	+7.1	+28.6	+32.8	+3.8	+15.9
Law enforcement												
T1	2.66	2.92	2.80	2.53	2.89	2.84	3.12	3.11	2.96	2.88	2.98	2.83
T2	3.04	3.22	2.94	2.46	2.87	2.98	3.78	3.49	3.55	3.07	3.02	3.75
% Change	+14.3	+10.3	+5.0	−2.8	−0.7	+4.9	+21.2	+12.2	+19.9	+6.6	+1.3	+32.5

Abbreviation: PCP, primary care physician.

^a^ Openness to firearm storage practices were scored on a scale of 1 to 5, with 1 indicating not at all open; 2, slightly open; 3, moderately open; 4, very open; and 5, extremely open.

The MANOVA indicated a significant interaction among time, messenger profession, gun-friendly text, and home protection text across all outcomes (Wilks’ λ *F* = 2.09, *P* = .01, _p_η^2^ = 0.040) (Table 3). Repeated-measures post hoc ANOVAs indicated the interaction was statistically significant for only 1 of the outcomes: storing firearms away from home with a trusted family member or friend (*F* = 5.42, *P* = .005; _p_η^2^ = 0.030). Further probing of this interaction indicated that several combinations of messenger and message content yielded positive changes in openness to this storage option (Table 4).

**Table 3.** Repeated-Measures Post Hoc Analyses of Variance Examining the Interactions of Time and Messaging Conditions Across All 7 Firearm Storage Options

Firearm storage option	*F*	*P* value	Cohen d
Time × messenger × gun friendly × home protection			
Unloaded	2.50	.08	.24
Separate from ammunition	2.96	.05	.26
Locked location	0.68	.51	.13
Locking device	0.28	.76	.09
Friend or family	5.42	.005	.35
Retailer	1.06	.35	.16
Law enforcement	2.80	.06	.26
Time × messenger × gun friendly			
Unloaded	2.81	.06	.26
Separate from ammunition	4.76	.009	.33
Locked location	0.97	.38	.14
Locking device	6.02	.002	.38
Friend or family	2.28	.10	.23
Retailer	0.72	.49	.13
Law enforcement	0.66	.52	.13
Time × messenger × home protection			
Unloaded	2.20	.11	.22
Separate from ammunition	3.23	.04	.27
Locked location	12.11	<.001	.52
Locking device	4.43	.010	.33
Friend or family	0.55	.58	.11
Retailer	2.47	.09	.24
Law enforcement	3.64	.03	.29
Time × gun friendly × home protection			
Unloaded	1.82	.18	.14
Separate from ammunition	0.00	.98	.00
Locked location	0.86	.36	.09
Locking device	3.73	.05	.20
Friend or family	1.75	.19	.14
Retailer	0.25	.62	.06
Law enforcement	0.50	.48	.06

**Table 4.** Time 1 and Time 2 Levels of Willingness to Use Specific Firearm Storage Option for Interactions Observed to Be Significant in Repeated-Measures Post Hoc Analyses of Variance

Firearm storage option	Mean (SE) [95% CI]		% Change
	Time 1	Time 2	
Separate from ammunition			
Primary care × gun friendly	2.72 (0.18) [2.36-3.09]	3.35 (0.19) [2.97-3.72]	+23.2
Combat controller × no gun friendly	2.87 (0.19) [2.50-3.24]	3.41 (0.19) [3.02-3.79]	+18.8
Primary care × no home protection	3.22 (0.18) [2.86-3.57]	3.72 (0.19) [3.35-4.08]	+15.5
Combat controller × home protection	3.00 (0.20) [2.61-3.40]	3.53 (0.21) [3.12-3.94]	+17.7
Locked location			
Primary care × no home protection	2.88 (0.19) [2.52-3.25]	3.53 (0.21) [3.12-3.94]	+22.6
Security forces × home protection	3.37 (0.20) [2.98-3.76]	3.93 (0.19) [3.56-4.31]	+16.6
Combat controller × no home protection	3.27 (0.17) [2.94-3.60]	4.10 (0.16) [3.78-4.42]	+25.4
Combat controller × home protection	2.88 (0.21) [2.47-3.29]	3.90 (0.20) [3.50-4.30]	+35.4
Locking device			
Primary care × gun friendly	2.93 (0.18) [2.58-3.28]	3.73 (0.18) [3.38-4.08]	+27.3
Security forces × no gun friendly	3.21 (0.16) [2.90-3.51]	3.88 (0.15) [3.57-4.18]	+20.9
Combat controller × no gun friendly	2.77 (0.18) [2.41-3.12]	3.55 (0.18) [3.19-3.90]	+28.2
Primary care × no home protection	3.10 (0.17) [2.76-3.44]	4.25 (0.17) [3.91-4.59]	+37.1
Security forces × no home protection	2.64 (0.16) [2.32-2.95]	3.08 (0.16) [2.77-3.39]	+16.7
Security forces × home protection	3.58 (0.19) [3.21-3.95]	3.96 (0.19) [3.60-4.33]	+10.6
Combat controller × no home protection	3.32 (0.16) [3.01-3.64]	3.95 (0.16) [3.64-4.26]	+19.0
Family or friend			
Primary care × gun friendly × no home protection	2.93 (0.26) [2.42-3.45]	3.80 (0.27) [3.27-4.32]	+29.7
Primary care × gun friendly × home protection	2.22 (0.28) [1.68-2.77]	2.85 (0.28) [2.30-3.40]	+28.4
Security forces × no gun friendly × home protection	3.11 (0.23) [2.64-3.57]	3.66 (0.24) [3.19-4.12]	+17.7
Security forces × gun friendly × no home protection	2.51 (0.24) [2.03-3.00]	3.09 (0.25) [2.61-3.57]	+23.1
Combat controller × no gun friendly × no home protection	2.91 (0.22) [2.47-3.34]	3.74 (0.22) [3.31-4.18]	+28.5
Combat controller × gun friendly × home protection	2.69 (0.27) [2.16-3.22]	3.28 (0.27) [2.74-3.81]	+21.9
Law enforcement			
Primary care × no home protection	2.70 (0.19) [2.33-3.07]	3.17 (0.21) [2.75-3.58]	+17.4
Security forces × home protection	3.15 (0.20) [2.76-3.55]	3.77 (0.23) [3.33-4.22	+19.7

Given the general lack of significant effects for the interaction of time with all 3 messaging conditions, we examined each 2-way interaction of time with each messaging condition. No interaction terms were significantly associated with willingness to store firearms unloaded or away from home at a firearm retailer. Descriptions of significant interaction terms for the other outcomes are described below.

The messenger × gun-friendly and messenger × home protection interactions were associated with willingness to store firearms separately from ammunition. Significant increases in willingness were observed when the primary care physician was paired with gun-friendly text (increase of 23.2%) or not paired with home protection text (increase of 15.5%) and when the combat controller was paired with home protection text (increase of 17.7%) or not paired with gun-friendly text (increase of 18.8%).

The messenger × home protection interaction was associated with willingness to store firearms in a locked location (Table 4). Significant increases in willingness to store firearms in a locked location were observed when the primary care physician or combat controller were (16.6% increase for primary care physician and 35.4% increase for combat controller) and were not (22.6% increase for primary care physician and 25.4% increase for combat controller) paired with home protection text.

Both the messenger × gun-friendly text and messenger × home protection text interactions were associated with willingness to store firearms with a locking device (Table 4). Significant increases were observed when the primary care physician was paired with gun-friendly text (increase of 27.3%) or not paired with home protection text (increase of 37.1%), when security forces was paired with home protection text (increase of 10.6%) or not paired with gun-friendly text (increase 20.9%) or home protection text (increase of 16.7%), and when the combat controller was not paired with gun-friendly text (increase 28.2%) or home protection text (increase 19.0%).

Finally, only the interaction of messenger profession × home protection text was associated with willingness to store firearms away from home with law enforcement (Table 4). Specifically, increased willingness was observed when the primary care physician was not paired with home protection text (increase of 17.4%) and when security forces was paired with home protection text (increase of 19.7%).

## Discussion

In this comparative effectiveness study, we aimed to build on the literature regarding safe firearm storage messaging by leveraging an experimental design to test the association of messenger profession, gun-friendly text, and home protection text on openness to various firearm storage practices among firearm-owning US service members. We were interested in the association of exposure to specific messages on openness to 4 at-home firearm storage practices (unloaded, separately from ammunition, in a locked location, and with a locking device) and 3 away-from-home firearm storage practices (with a family member or friend, at a firearm retailer, or at a law enforcement agency). Participants were exposed to only a single message a single time, and each condition overlapped in aspects of the imagery and text; however, we anticipated that differences in messenger profession and message content would nonetheless exhibit differential associations with subsequent shifts in willingness.

Before exposure to messaging, mean scores on willingness to store firearms using a locking device were higher than those for other storage approaches. This finding suggests that there may be value in initially emphasizing locking devices to increase buy-in, with the hope of increased willingness to use additional approaches after adopting a locking device. The magnitude and nature of shifts in willingness after exposure to the messages varied. In this article, we focus on results with *P* < .01. The omnibus within-group analysis suggested that there was an overall change in willingness to adopt safe firearm storage practices over time across the 3 message factors. Univariate analyses suggested that this was primarily driven by changes in willingness to store firearms outside the home with a friend or family member. Significant increases in willingness emerged when both the gun-friendly and home protection messages were paired with any of the messengers, when the gun-friendly (but not home protection) message was paired with primary care and security forces, and when the home protection (but not gun-friendly) message was paired with security forces. This was the only significant finding specific to outside-the-home storage, indicating that prompting outside-of-home storage could prove difficult. Given the success of the combination of gun-friendly and home protection messages regardless of messenger, these preliminary results suggest that content may be more impactful than messenger profession for this particular outcome.

When examining the results of the 2-way interactions, the role of the messenger appeared more pronounced. Messenger profession moderated the association of the gun-friendly and home protection texts with multiple in-home storage practices. With respect to locking devices, we saw increased willingness when the primary care physician was paired with a gun-friendly message, when security forces and combat controller were not paired with the gun-friendly message, when any messenger was not paired with the home protection message, and when security forces was paired with home protection. For the use of locked locations, all messengers yielded increased willingness when paired with the home protection message and combat controller also did when not paired with the home protection message. With respect to storing firearms separately from ammunition, both primary care and combat controller increased willingness when paired with the gun-friendly text and combat controller also increased willingness when not paired with the gun-friendly text.

Overall, firearm-owning service members appear more open to adopting in-home than outside-of-home firearm storage practices for suicide prevention. This finding may indicate that promoting in-home storage would be more impactful initially and highlights the importance of finding a path toward making outside-of-home storage more palatable. The overall pattern of the findings also indicates that messages delivered by the security forces and combat controller messengers yielded positive change under a broader array of message content options. This finding does not mean that the use of physicians can never be beneficial in broad public health messaging, but it does highlight that there may be more message flexibility when certain messengers are used.

At the univariate level, there is modest evidence to suggest the risk of iatrogenic effects is lower for the security forces and combat controller than for the primary care physician. Findings indicating that physicians may not be optimal messengers for broad public health messaging efforts, however, do not imply that clinical interactions focused on safe firearm storage are ineffective.

Several prior studies^16,17,18^ have identified law enforcement as consistently being rated the most credible messenger on safe firearm storage. Although not synonymous with law enforcement, security forces perform a similar function within the military context and may be seen as particularly credible given their universal training in firearm use and the fact that they are more likely than other military personnel to carry firearms while on a military installation. Prior work had simply asked individuals to rank-order potential messengers. These findings represent the first experimental evidence supporting the potential value of law enforcement in safe storage messaging. This support, however, differs from the survey findings in that there was less evidence directly pointing to security forces as the clear preference.

Of note, participants viewed a single message—one that was far more similar than different across conditions—a single time, and changes in willingness to use specific storage practices were assessed in the moments immediately after exposure. Thus, the dosage of the intervention was low, variation in the messages was slight, the context of the intervention delivery differed from normal, and the period during which attitudinal shifts were maintained was minimal. Complementary research on behavioral economics–informed nudges has shown promise in prompting suicide prevention–related behavior change through small messaging changes.^21,22,23^

### Strengths and Limitations

Firearm suicide decedents are less likely than those who used other methods to have received behavioral health care or to have survived a prior suicide attempt.^24^ We must develop interventions that reach firearm owners of unknown suicide risk through channels that are likely to resonate. A strength of this study is the potential scalability of this intervention, which highlights the importance of future research that considers other components and formats of messaging.

However, several limitations are worth noting. Our outcome variable was willingness to adopt firearm storage practices rather than actual storage changes. Nonetheless, our experimental design represents a meaningful initial step in the development and testing of safe storage messaging. Our analysis was underpowered to examine demographic moderators (eg, gender) that might clarify for whom specific aspects of the message were vital. No single message or messenger will likely prove universally compelling. Despite these limitations, we believe this work provides useful information regarding public health strategies for preventing firearm suicide.

## Conclusions

The fact that the messaging yielded change in a sample of US service members—a group shown to underreport thoughts of suicide and avoid behavioral health care^25^—highlights that messaging has the potential to serve populations otherwise difficult to reach. Our findings cannot be generalized beyond the military; however, prior research^16^ has shown that civilian and military firearm owners tend to endorse similar ratings for potential messengers. The findings highlight the potential utility of a scalable intervention—visual messages on safe firearm storage—in prompting behavior changes.

## Notes

Supplement. **eAppendix.** Supplemental Information on Study Design, Survey Responses, and Sample Weighting/Calibrating Procedures **eFigure 1.** Stimulus Image for Primary Care Physician, Non-Gun Friendly, Non-Home Protection **eFigure 2.** Stimulus Image for Primary Care Physician, Gun Friendly, Non-Home Protection **eFigure 3.** Stimulus Image for Primary Care Physician, Gun Friendly, Home Protection **eFigure 4.** Stimulus Image for Primary Care Physician, Non-Gun Friendly, Home Protection **eFigure 5.** Stimulus Image for Security Forces, Non-Gun Friendly, Non-Home Protection **eFigure 6.** Stimulus Image for Security Forces, Gun Friendly, Non-Home Protection **eFigure 7.** Stimulus Image for Security Forces, Gun Friendly, Home Protection **eFigure 8.** Stimulus Image for Security Forces, Non-Gun Friendly, Home Protection **eFigure 9.** Stimulus Image for Combat Controller, Non-Gun Friendly, Non-Home Protection **eFigure 10.** Stimulus Image for Combat Controller, Gun Friendly, Non-Home Protection **eFigure 11.** Stimulus Image for Combat Controller, Gun Friendly, Home Protection **eFigure 12.** Stimulus Image for Combat Controller, Non-Gun Friendly, Home Protection
