# Supplementary material for: Evaluation of Safe Firearm Storage Messaging in a Sample of Firearm-Owning US Military Service Members
Source: JAMA Netw Open. 2022 Oct 11;5(10):e2235984. doi: 10.1001/jamanetworkopen.2022.35984 (PMC9554700; doi:10.1001/jamanetworkopen.2022.35984)
Supplement: Supplement. — eAppendix. Supplemental Information on Study Design, Survey Responses, and Sample Weighting/Calibrating Procedures eFigure 1. Stimulus Image for Primary Care Physician, Non-Gun Friendly, Non-Home Protection eFigure 2. Stimulus Image for Primary Care Physician, Gun Friendly, Non-Home Protection eFigure 3. Stimulus Image for Primary Care Physician, Gun Friendly, Home Protection eFigure 4. Stimulus Image for Primary Care Physician, Non-Gun Friendly, Home Protection eFigure 5. Stimulus Image for Security Forces, Non-Gun Friendly, Non-Home Protection eFigure 6. Stimulus Image for Security Forces, Gun Friendly, Non-Home Protection eFigure 7. Stimulus Image for Security Forces, Gun Friendly, Home Protection eFigure 8. Stimulus Image for Security Forces, Non-Gun Friendly, Home Protection eFigure 9. Stimulus Image for Combat Controller, Non-Gun Friendly, Non-Home Protection eFigure 10. Stimulus Image for Combat Controller, Gun Friendly, Non-Home Protection eFigure 11. Stimulus Image for Combat Controller, Gun Friendly, Home Protection eFigure 12. Stimulus Image for Combat Controller, Non-Gun Friendly, Home Protection [file jamanetwopen-e2235984-s001.pdf]

Supplementary Online Content

Anestis MD, Bryan CJ, Capron DW, Bryan AO. Evaluation of safe firearm storage messaging in sample of firearm-owning US military service members. *JAMA Netw Open*. 2022;5(10):e2235984. doi:10.1001/jamanetworkopen.2022.35984

eAppendix. Supplemental Information on Study Design, Survey Responses, and Sample Weighting/Calibrating Procedures

eFigure 1. Stimulus Image for Primary Care Physician, Non-Gun Friendly, Non-Home Protection

eFigure 2. Stimulus Image for Primary Care Physician, Gun Friendly, Non-Home Protection

eFigure 3. Stimulus Image for Primary Care Physician, Gun Friendly, Home Protection

eFigure 4. Stimulus Image for Primary Care Physician, Non-Gun Friendly, Home Protection

eFigure 5. Stimulus Image for Security Forces, Non-Gun Friendly, Non-Home Protection

eFigure 6. Stimulus Image for Security Forces, Gun Friendly, Non-Home Protection

eFigure 7. Stimulus Image for Security Forces, Gun Friendly, Home Protection

eFigure 8. Stimulus Image for Security Forces, Non-Gun Friendly, Home Protection

eFigure 9. Stimulus Image for Combat Controller, Non-Gun Friendly, Non-Home Protection

eFigure 10. Stimulus Image for Combat Controller, Gun Friendly, Non-Home Protection

eFigure 11. Stimulus Image for Combat Controller, Gun Friendly, Home Protection

eFigure 12. Stimulus Image for Combat Controller, Non-Gun Friendly, Home Protection

This supplementary material has been provided by the authors to give readers additional information about their work.

© 2022 Anestis MD et al. *JAMA Network Open.*

eAppendix. Supplemental Information on Study Design, Survey Responses, and Sample Weighting/Calibrating Procedures

Study design.

Our team opted to assess three specific factors within the visual messages: the profession of the messenger, the inclusion of "gun friendly" text, and the inclusion of text validating the drive for home protection. The rationale for these decisions are as follows:

Profession – We opted to consider the profession of the messenger to align with a series of survey-based studies published by our team and others in which individuals – servicemembers and civilians – have been asked to rank order potential messengers on safe firearm storage based upon credibility. The general pattern of the findings from those studies indicated that law enforcement (a parallel for Security Forces) was consistently seen as a highly credible messenger whereas medical professionals were often seen as having limited credibility on this issue (with respect to broad public health messaging). We included a Combat Controller messenger as a way to include an individual with clear aptitude with firearms and who we assumed would be unlikely to be seen as having an anti-firearm agenda.

Gun Friendly Text – We opted to consider the value of including this based upon prior research. Extant literature has indicated that validating the perspective of firearm owners is a key component to messaging.

Home Protection Text – We opted to consider the value of home protection text based upon two reasons. First, home protection is the most commonly endorsed reason for firearm ownership and, in our prior work (e.g. Project Safe Guard), we have found that owning firearms for protective reasons prompts resistance to safe firearm storage, presumably because individuals feel the need for quick access in case of home invasion. Second, in our discussions with the Defense Suicide Prevention Office about their recent focus groups on this topic, they indicated that validating this need while including statistics that support reconsidering the risk ratio was endorsed as particularly important among service members in messaging on safe storage.

Sample responses.

76% of individuals in KP who were sent the survey responded. 28% of those individuals met inclusion criteria by confirming both their military affiliation and current firearm ownership.

Opt-in samples included additional proprietary panels run by IPSOS as well as panels administered by trusted IPSOS partners. In each case, a router approach was used wherein an invitation-link would take participants to their homepage at the panel website where they could pick surveys to participate in based upon their perceived eligibility. Invitations are not sent for specific surveys – rather panel members are allocated into surveys the system identifies them as being qualified to participate in, with qualification based on inclusion criteria further determined based upon responses to the relevant survey items (e.g. firearm ownership status). This approach, adopted by IPSOS in 2021, renders completion rate impossible to calculate. As such, a 3% qualification rate is the more relevant reportable variable.

Median completion time for the study was 24 minutes. Upon completion, qualified KP respondents received an entry into the KP sweepstakes.

© 2022 Anestis MD et al. *JAMA Network Open.*

Sample weighting.
The KnowledgePanel (KP) sample was weighted to represent individuals aged 18 and older within the current US military population. The US Census Bureau's Current Population Survey (CPS) is used to determine benchmarks for weighting. The benchmarks used included:

- Gender
- Age
- Race/Hispanic ethnicity
- Education
- Census region
- Household income
- Home ownership status
- Metropolitan area
- Hispanic origin

The weighting process for this particular study included two steps.

Step 1: Starting with a design weight of 1, current US military service respondents (regardless of their firearm ownership) were raked to the following geodemographic distributions of the 18 and over current US military service population. The needed benchmarks were obtained from KP profile data. The current US military service from KP was defined based on those who were either currently on active duty in the US Armed Forces or currently members of the Reserve or National Guard.

- Gender (Male, Female) by Age (18-29, 30-39, 40+)
- Race-Ethnicity (White/Non-Hispanic, Black/Non-Hispanic, Other or 2+ Races/Non-Hispanic, Hispanic)
- Census Region (Northeast, Midwest, South, West)
- Metropolitan Status (Metro, Non-Metro)
- Education (Some college or below, Bachelor or higher)
- Household Income (under $25k, $25k-$49,999, $50k-$74,999, $75k-$99,999, $100k or more)
- Calibration Variable 1 – Watch TV (< 3 hours/day, 3+ hours/day)
- Calibration Variable 2 – Internet for Personal Use (< 10 hours/week, 10+ hours/week)
- Calibration Variable 3 – Express Political/Community Opinions Online (Less than once a month or more often, Not at all)
- Calibration Variable 4 – Try new products (Not at all/Somewhat, A lot/Completely)

Step 2: The resulting weights were trimmed and scaled to add up to the total number of current US military respondents and qualified respondents. Qualified respondents were those who owned firearms.

Weighting, including calibration, was performed for screened completes (e.g. US military respondents regardless of their firearm ownership status). Qualified cases then carried appropriate weights for this subpopulation. Due to the small base size, the calibration benchmarks for opt-in panelists were derived from KP profile data, not the KP respondents from the study.

© 2022 Anestis MD et al. *JAMA Network Open.*

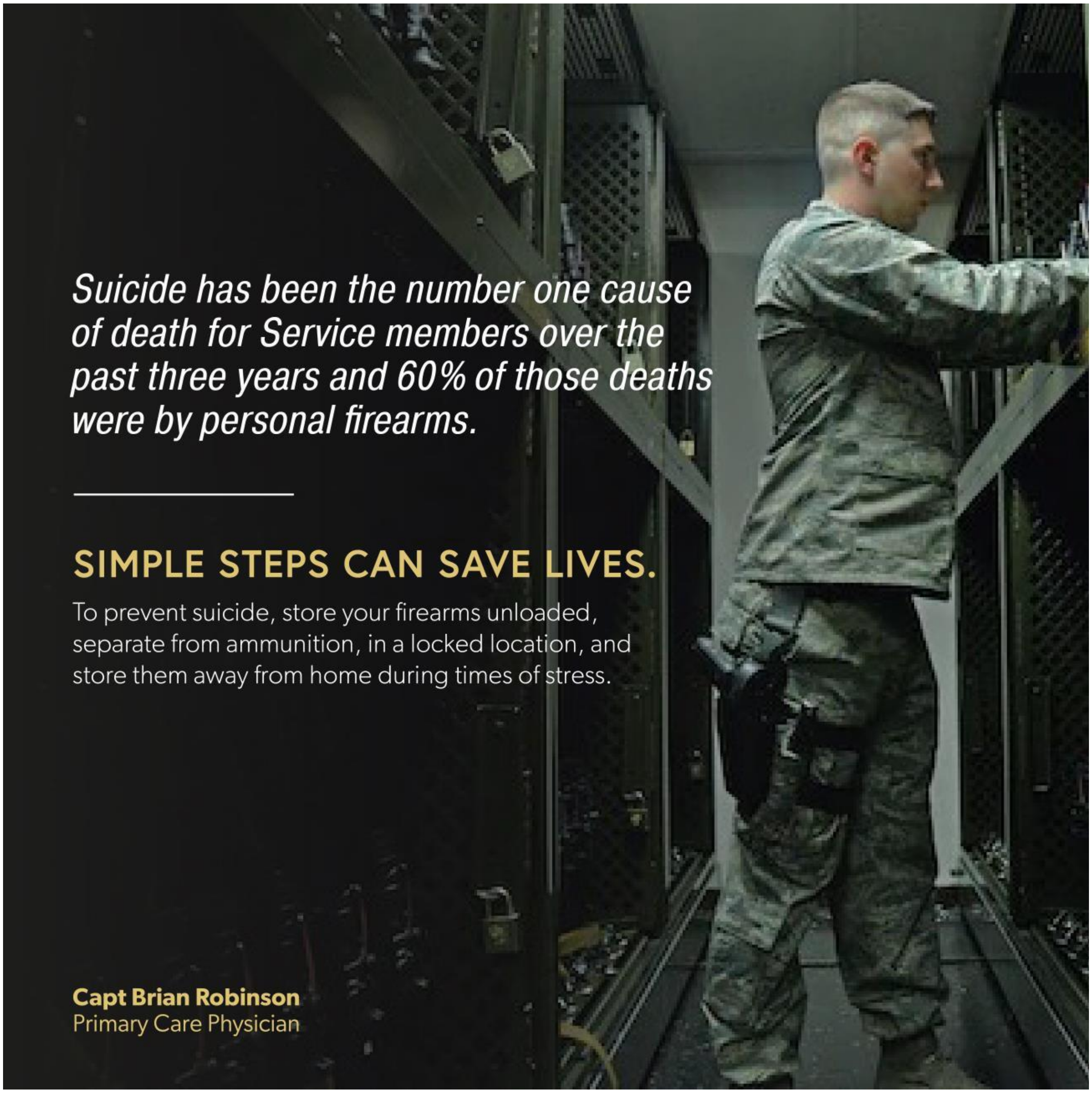

eFigure 1. Stimulus Image for Primary Care Physician, Non-Gun Friendly, Non-Home Protection

© 2022 Anestis MD et al. *JAMA Network Open.*

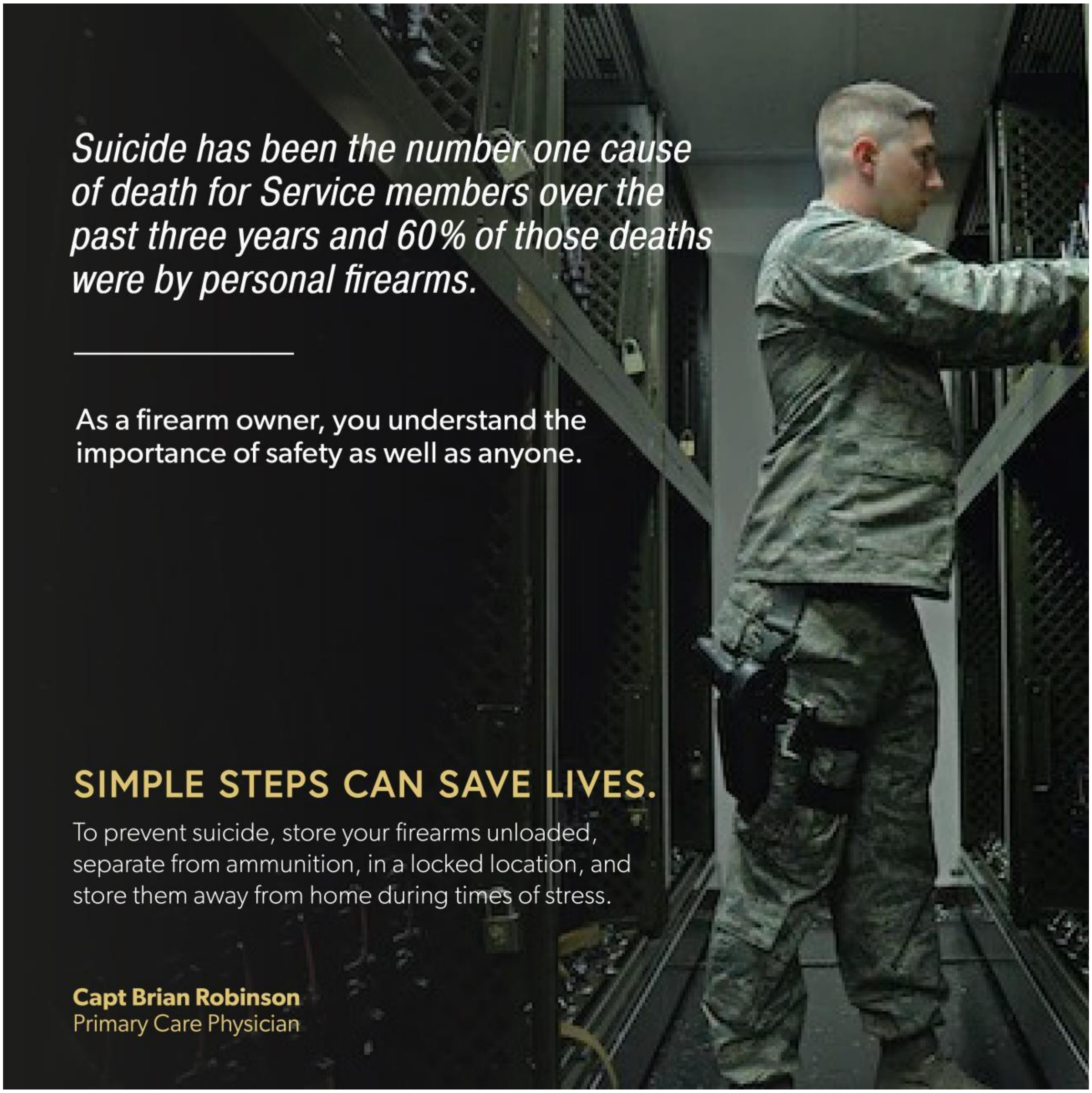

eFigure 2. Stimulus Image for Primary Care Physician, Gun Friendly, Non-Home Protection

© 2022 Anestis MD et al. *JAMA Network Open.*

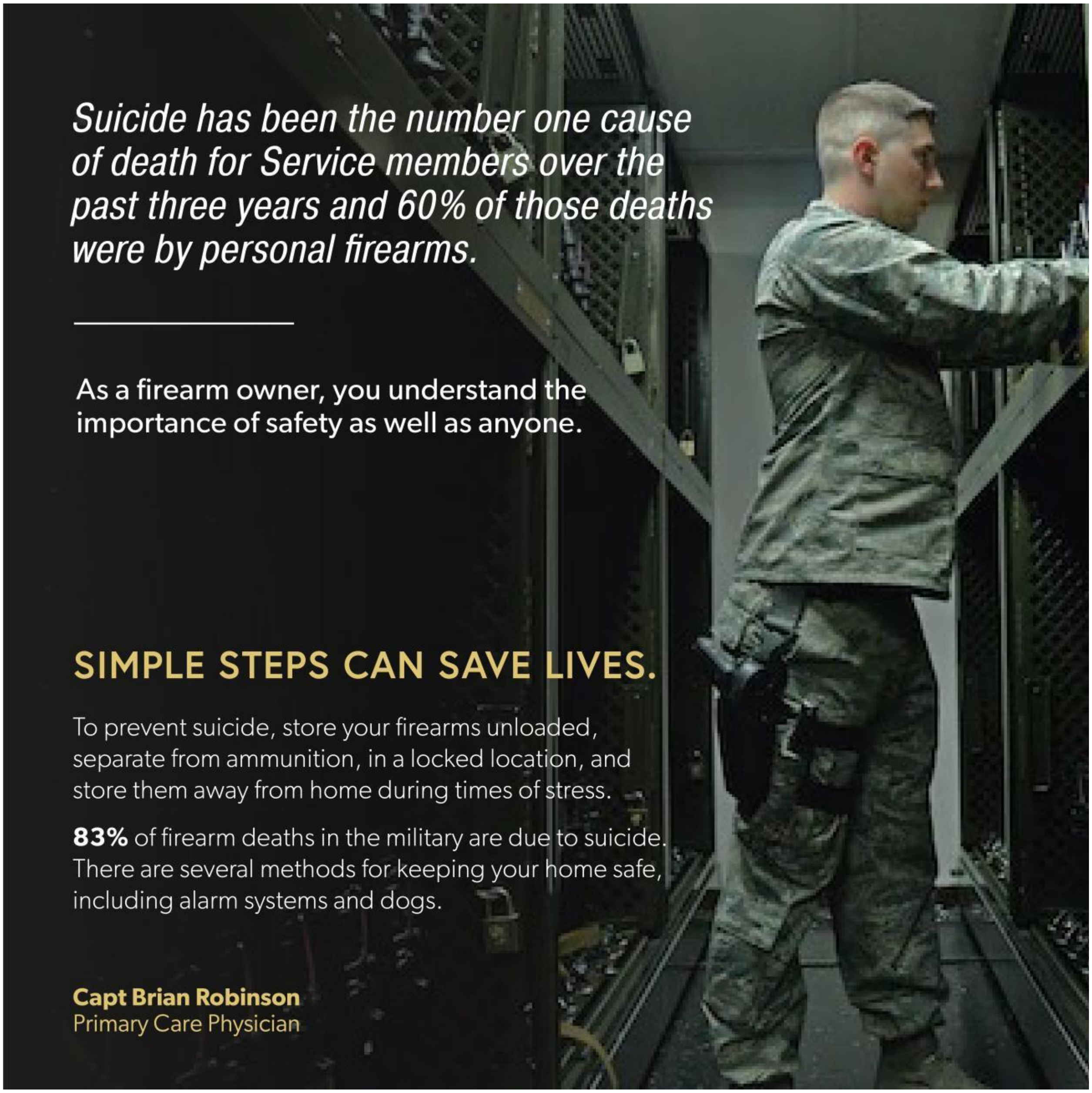

eFigure 3. Stimulus Image for Primary Care Physician, Gun Friendly, Home Protection

© 2022 Anestis MD et al. *JAMA Network Open.*

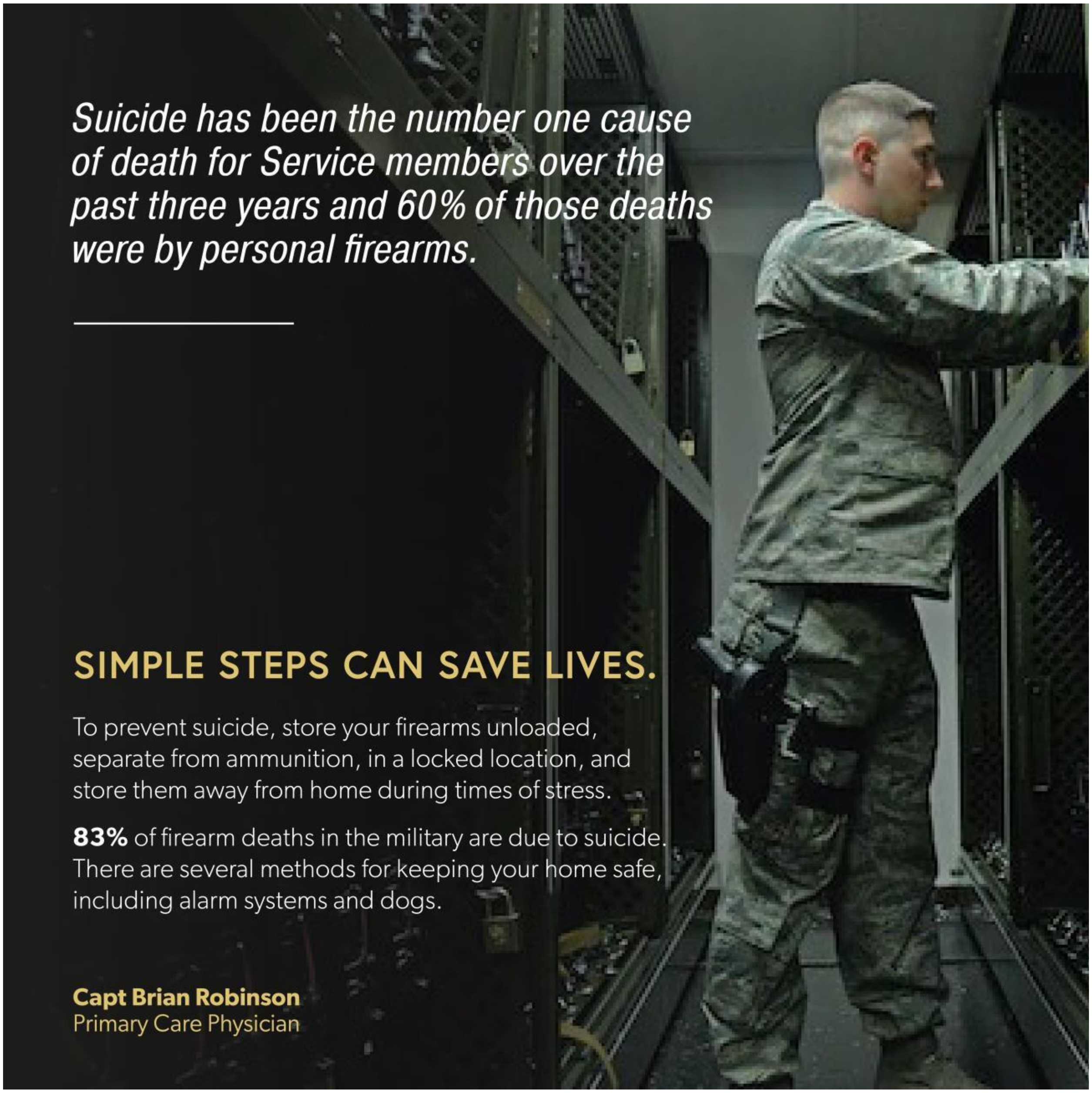

eFigure 4. Stimulus Image for Primary Care Physician, Non-Gun Friendly, Home Protection

© 2022 Anestis MD et al. *JAMA Network Open.*

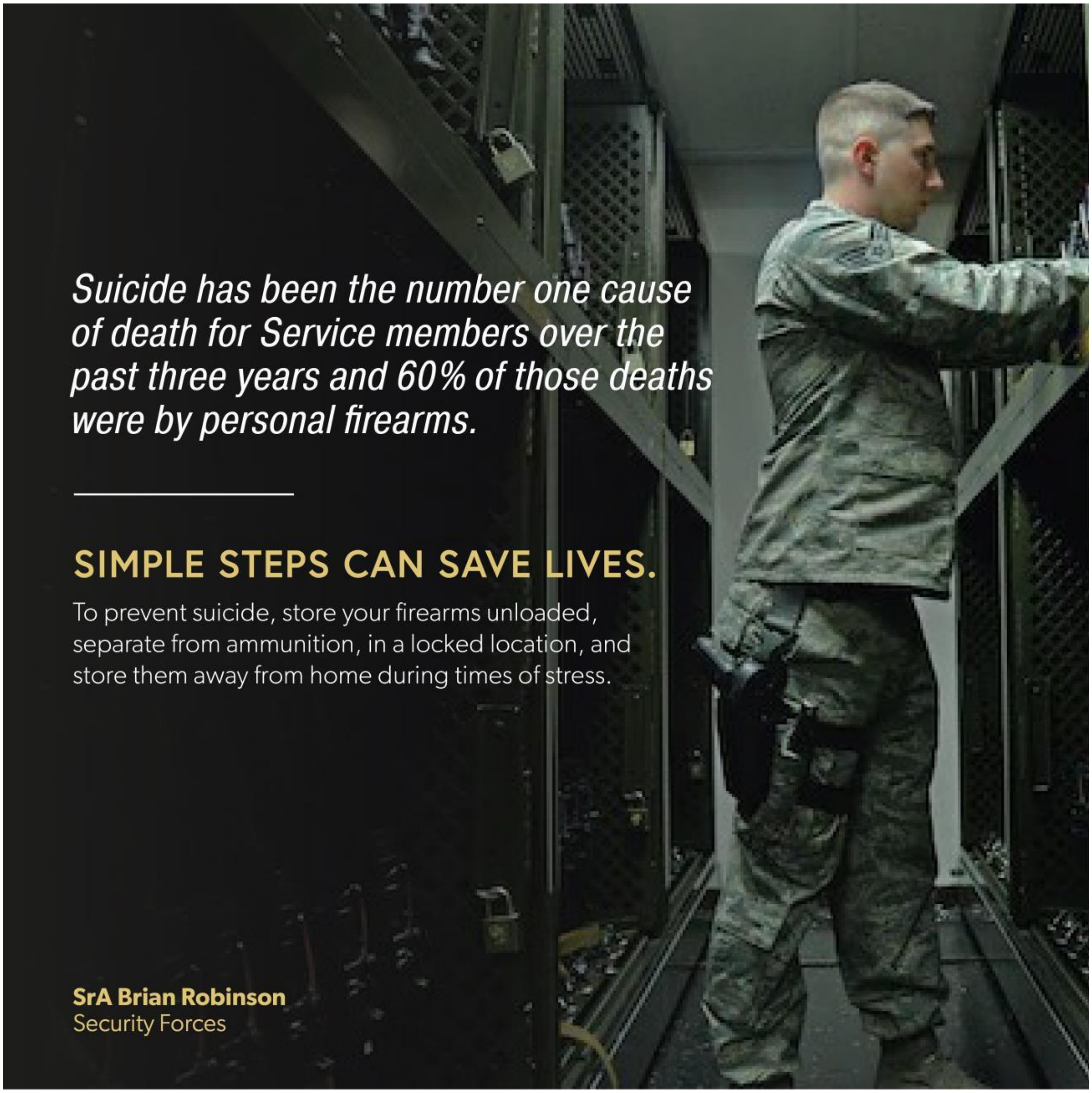

eFigure 5. Stimulus Image for Security Forces, Non-Gun Friendly, Non-Home Protection

© 2022 Anestis MD et al. *JAMA Network Open.*

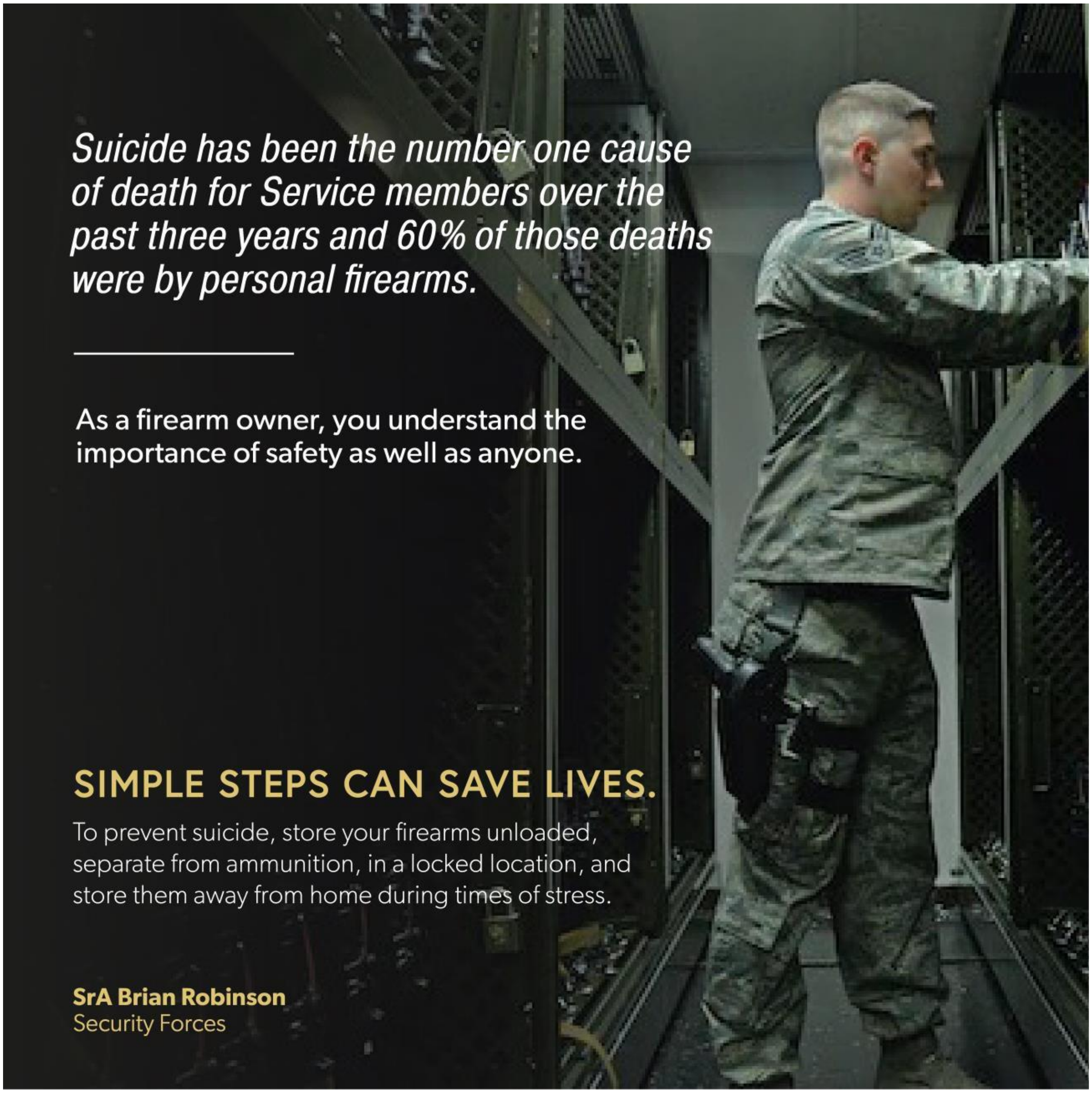

eFigure 6. Stimulus Image for Security Forces, Gun Friendly, Non-Home Protection

© 2022 Anestis MD et al. *JAMA Network Open.*

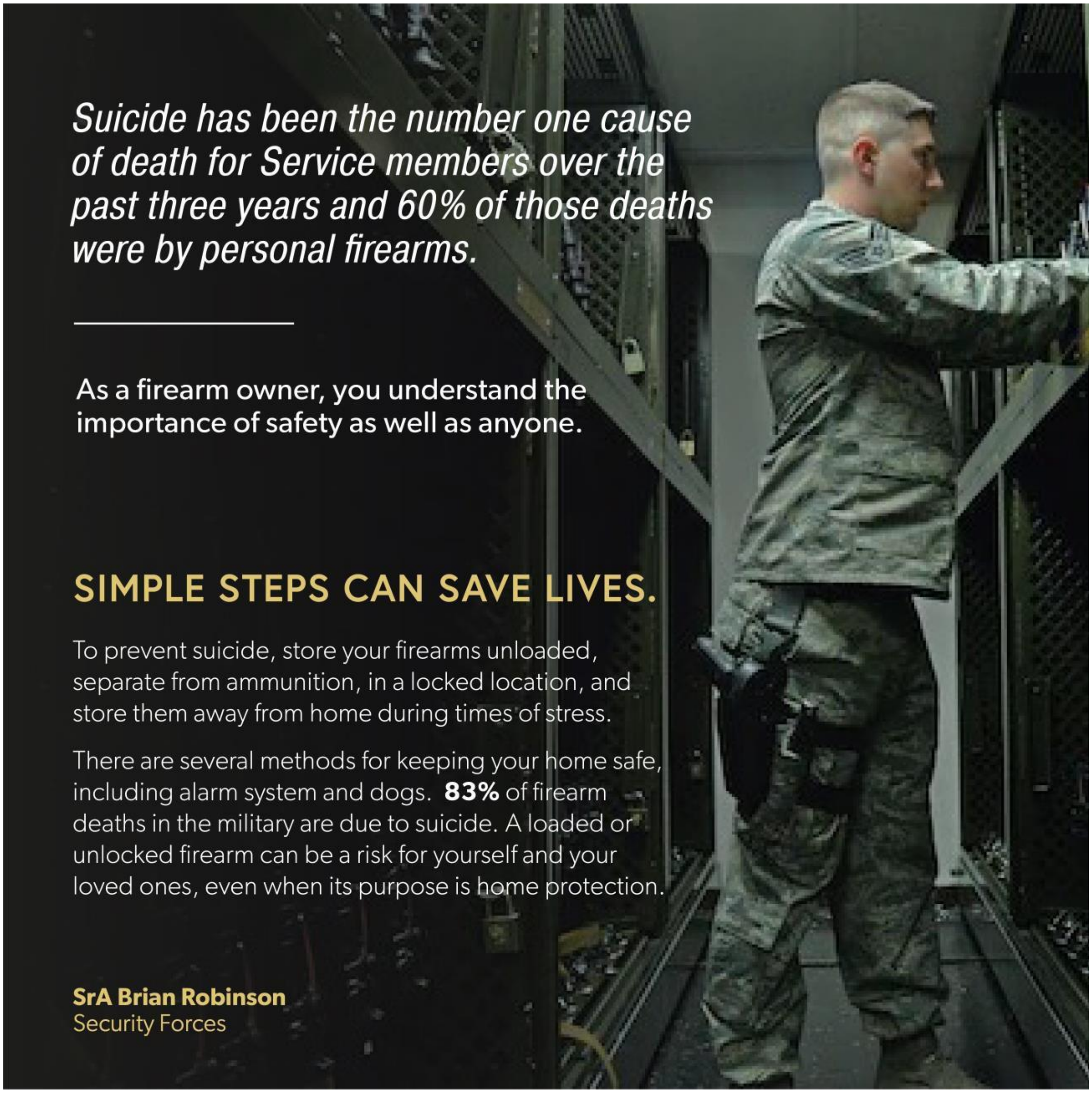

eFigure 7. Stimulus Image for Security Forces, Gun Friendly, Home Protection

© 2022 Anestis MD et al. *JAMA Network Open.*

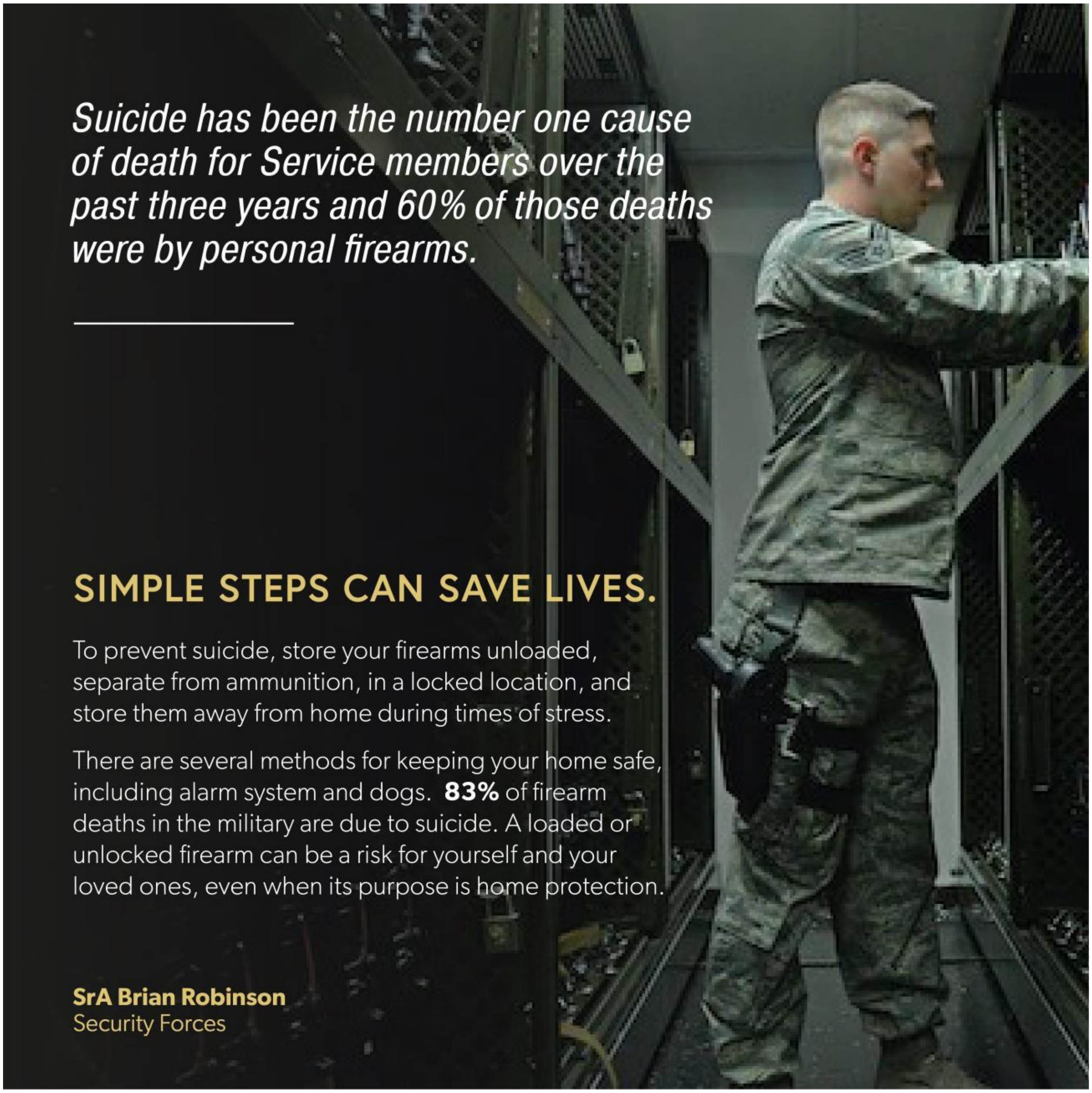

eFigure 8. Stimulus Image for Security Forces, Non-Gun Friendly, Home Protection

© 2022 Anestis MD et al. *JAMA Network Open.*

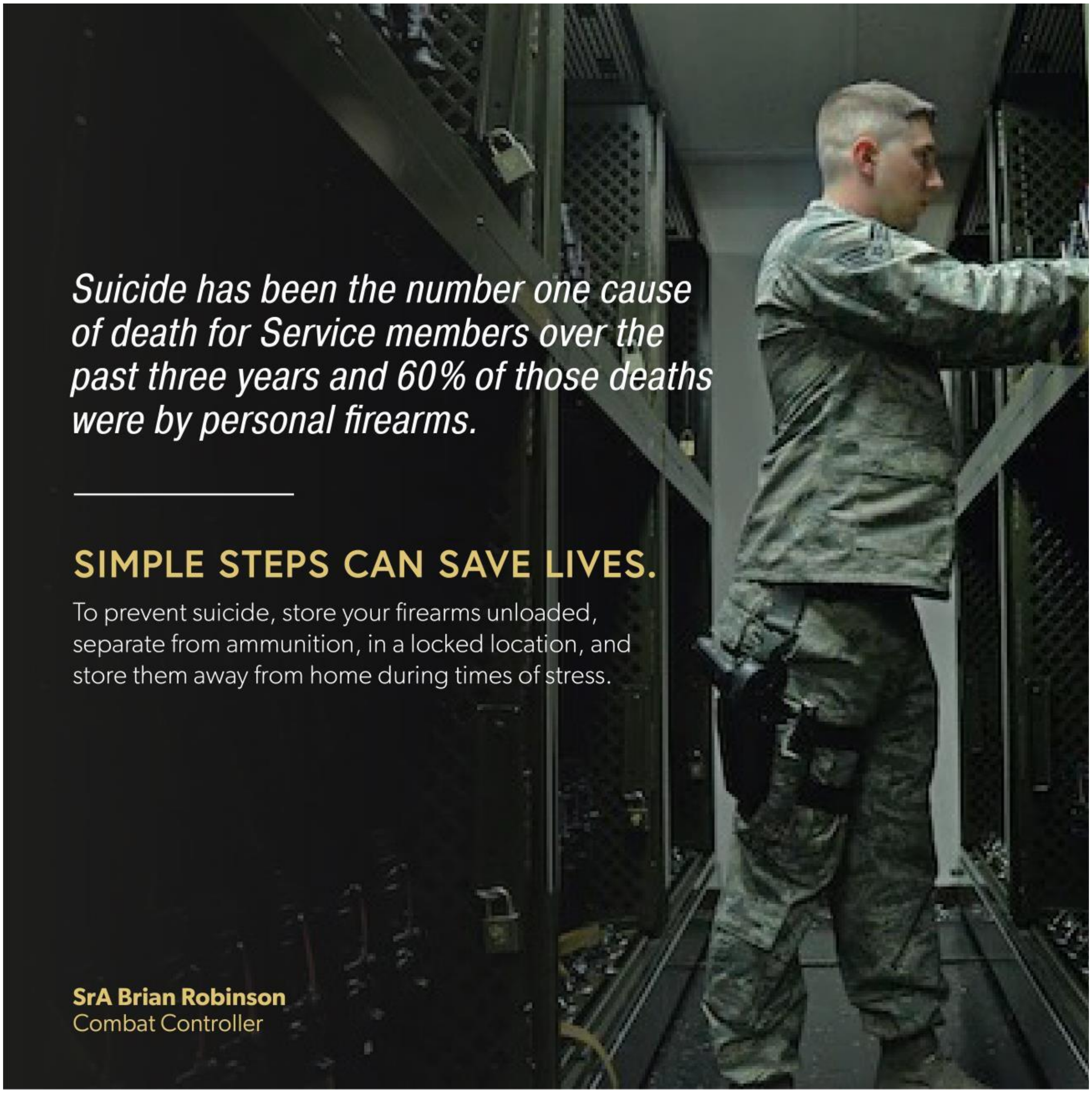

eFigure 9. Stimulus Image for Combat Controller, Non-Gun Friendly, Non-Home Protection

© 2022 Anestis MD et al. *JAMA Network Open.*

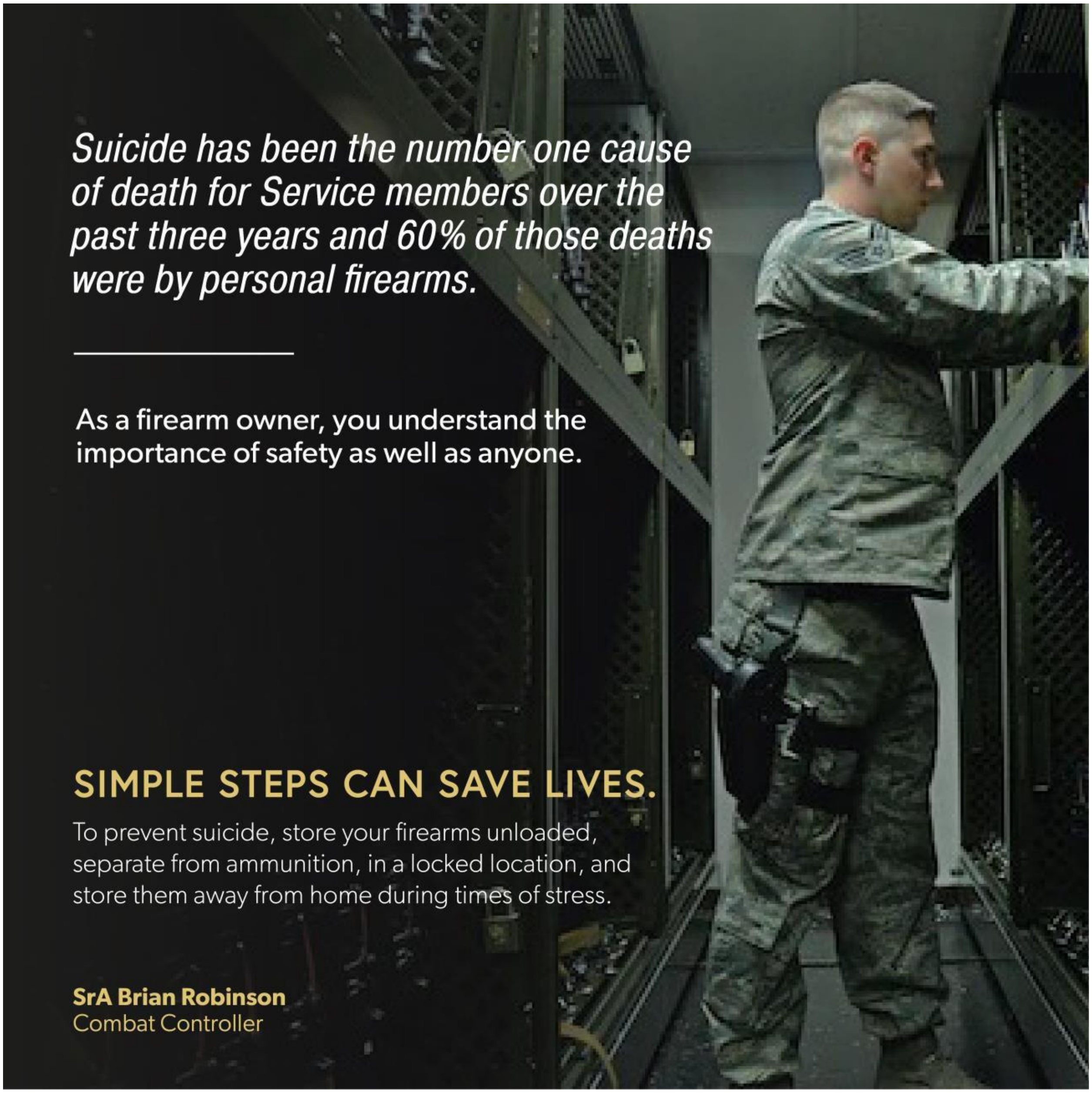

eFigure 10. Stimulus Image for Combat Controller, Gun Friendly, Non-Home Protection

© 2022 Anestis MD et al. *JAMA Network Open.*

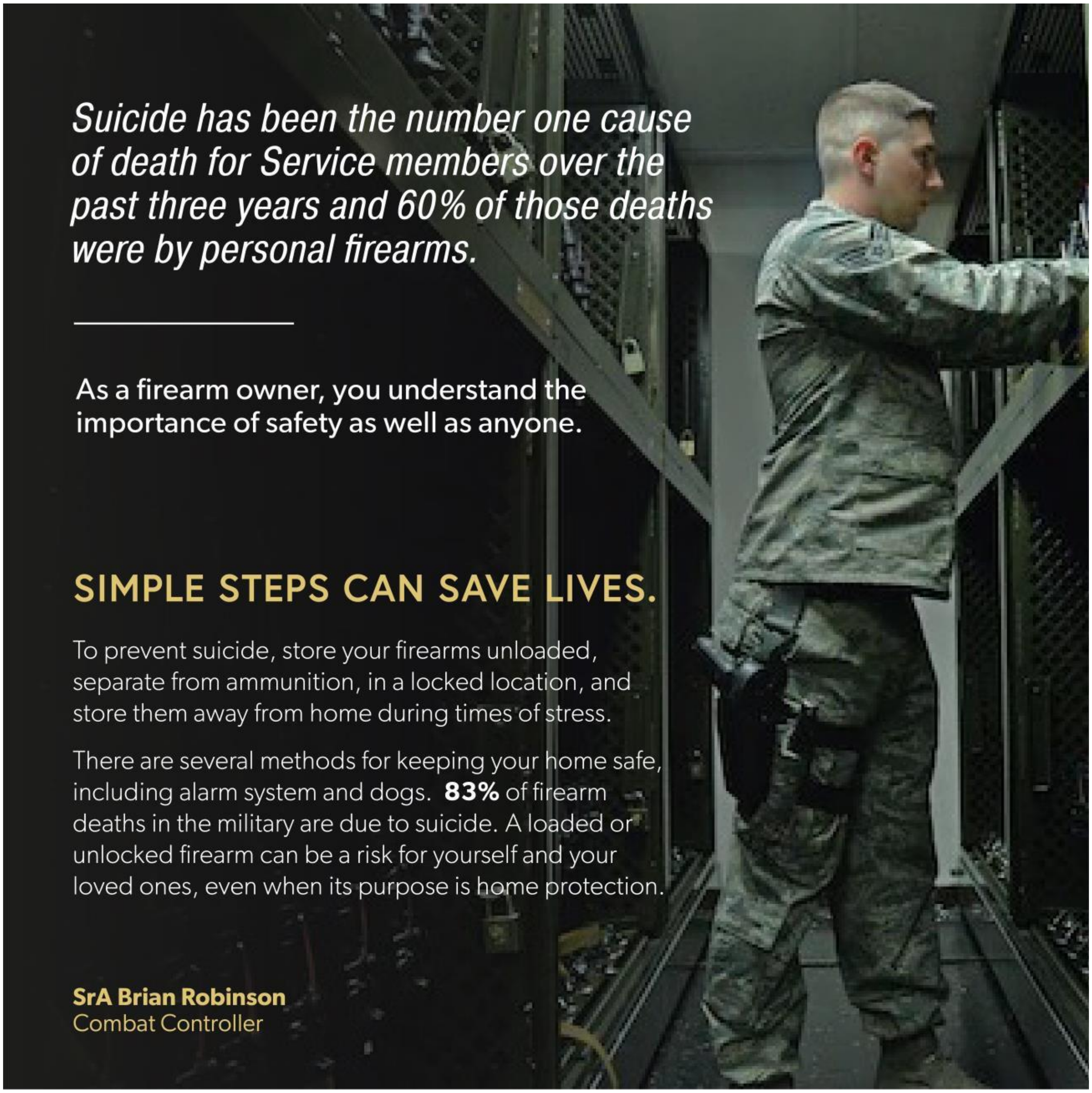

eFigure 11. Stimulus Image for Combat Controller, Gun Friendly, Home Protection

© 2022 Anestis MD et al. *JAMA Network Open.*

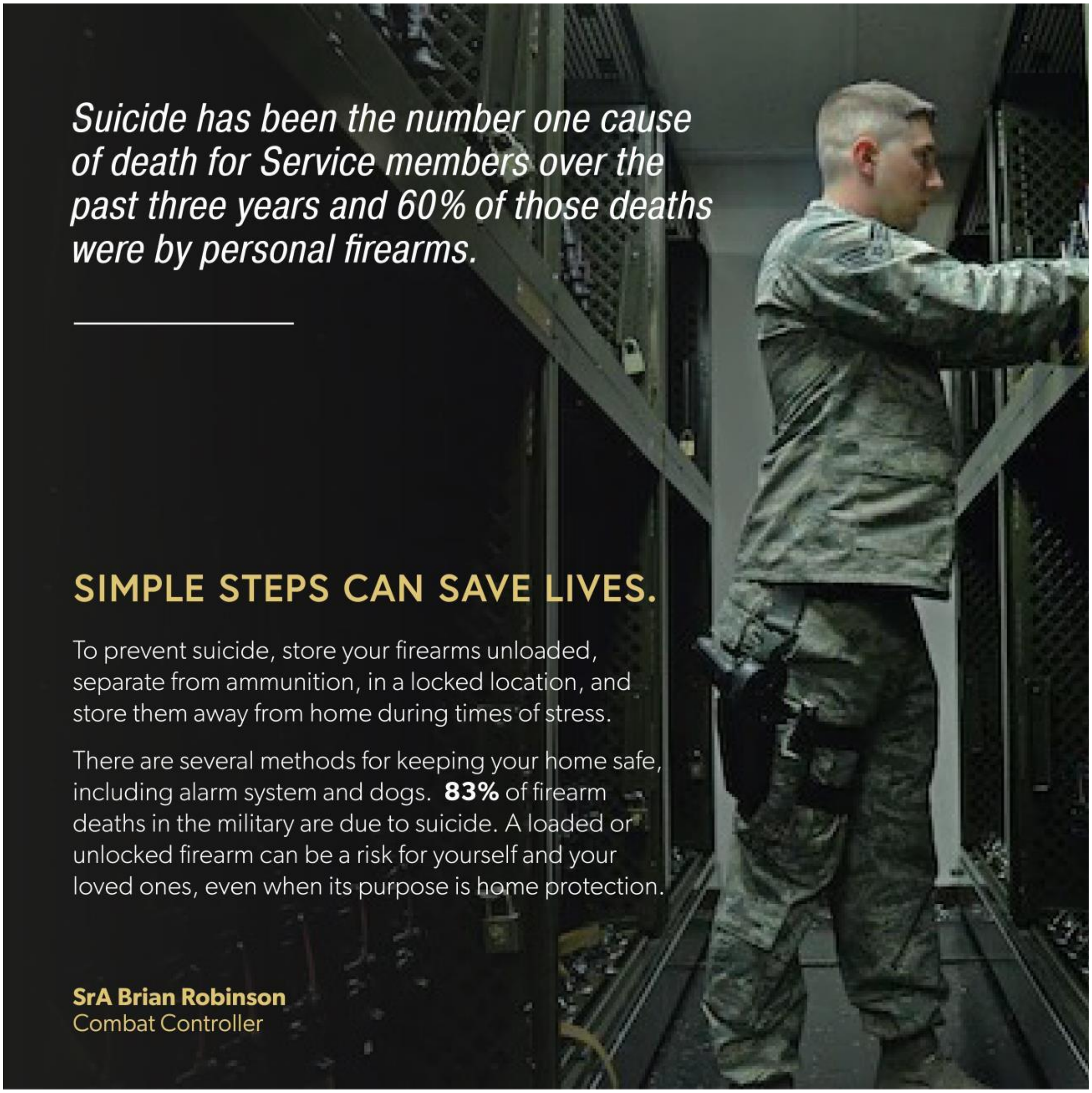

eFigure 12. Stimulus Image for Combat Controller, Non-Gun Friendly, Home Protection

© 2022 Anestis MD et al. *JAMA Network Open.*
